# A Portable Platform for Evaluation of Visual Performance in Glaucoma Patients

**DOI:** 10.1371/journal.pone.0139426

**Published:** 2015-10-07

**Authors:** Peter N. Rosen, Erwin R. Boer, Carolina P. B. Gracitelli, Ricardo Y. Abe, Alberto Diniz-Filho, Amir H. Marvasti, Felipe A. Medeiros

**Affiliations:** Visual Performance Laboratory, Department of Ophthalmology, University of California San Diego, La Jolla, United States of America; University of Houston, UNITED STATES

## Abstract

**Purpose:**

To propose a new tablet-enabled test for evaluation of visual performance in glaucoma, the PERformance CEntered Portable Test (PERCEPT), and to evaluate its ability to predict history of falls and motor vehicle crashes.

**Design:**

Cross-sectional study.

**Methods:**

The study involved 71 patients with glaucomatous visual field defects on standard automated perimetry (SAP) and 59 control subjects. The PERCEPT was based on the concept of increasing visual task difficulty to improve detection of central visual field losses in glaucoma patients. Subjects had to perform a foveal 8-alternative-forced-choice orientation discrimination task, while detecting a simultaneously presented peripheral stimulus within a limited presentation time. Subjects also underwent testing with the Useful Field of View (UFOV) divided attention test. The ability to predict history of motor vehicle crashes and falls was investigated by odds ratios and incident-rate ratios, respectively.

**Results:**

When adjusted for age, only the PERCEPT processing speed parameter showed significantly larger values in glaucoma compared to controls (difference: 243ms; P<0.001). PERCEPT results had a stronger association with history of motor vehicle crashes and falls than UFOV. Each 1 standard deviation increase in PERCEPT processing speed was associated with an odds ratio of 2.69 (P = 0.003) for predicting history of motor vehicle crashes and with an incident-rate ratio of 1.95 (P = 0.003) for predicting history of falls.

**Conclusion:**

A portable platform for testing visual function was able to detect functional deficits in glaucoma, and its results were significantly associated with history of involvement in motor vehicle crashes and history of falls.

## Introduction

Glaucoma is a neurodegenerative disease associated with progressive loss of retinal ganglion cells, characteristic optic nerve changes, and loss of visual function.[1] The disease is a leading cause of visual impairment and disability, and patients with glaucoma have been reported to be at an increased risk for motor vehicle collisions and falls, two important causes of morbidity and mortality in the elderly population.[2–12]

The routine evaluation of visual function in glaucoma is based on standard automated perimetry (SAP). However, the requirements for highly trained technicians, cost, complexity, and lack of portability of SAP testing preclude its general use for screening or evaluation of visual function loss in primary care settings or in underserved populations. In addition, SAP has a relatively weak ability to assess parameters that may be related to functional impairment from the disease, such as risk for motor vehicle collisions or falls, for example. In SAP, the ability to detect a static white-on-white peripheral visual stimulus at threshold is evaluated under optimal conditions of adaptation and testing. However, these artificial test conditions minimize potential distractions and may give unrealistic estimates of the amount of useful vision that is available to perform real work tasks.[13,14]

Previous studies have shown that the accuracy of detecting a peripheral stimulus decreases significantly when subjects are required to perform a simultaneous demanding foveal task.[15] The change in performance results from a narrowing of the spotlight of attention caused by the demands of the central task. These observations led to the concept of functional field of view, as introduced by Sanders[16]. In contrast to the traditional visual field test, an assessment of the functional field of view would better reflect the demands imposed on vision by everyday tasks, when subjects are frequently required to detect or monitor a peripheral stimulus, while simultaneously attending to a central task. Capitalizing on the concept of functional field of view, Ball and Owsley[17] developed the Useful Field of View test (UFOV, Visual Awareness, Inc, Chicago, IL) to assess visual processing speed under divided attention conditions. Several studies have suggested that the UFOV performs better than conventional perimetric tests in predicting risk of motor vehicle collisions and falls.[18,19] Although UFOV results may be affected by visual field sensitivity, the test has generally not been used for detection of field defects. In fact, the UFOV characteristics, such as use of suprathreshold stimuli at maximum contrast, would most likely make it unsuitable for this purpose or yield low diagnostic value.

In the current study, we propose and provide initial assessment of a test for evaluation of visual function in glaucoma, the PERformance-CEntered Portable Test (PERCEPT). Development of the test was based on the concept of increasing visual task difficulty to improve the sensitivity for uncovering visual deficits in glaucoma. Increasing visual performance load was accomplished by combining spatial, temporal and contrast components into a single test, requiring subjects to perform a demanding, time-constrained, dual visual task at low contrast. Recent studies have demonstrated that macular retinal ganglion cell loss in glaucoma seems to occur at the same proportion as peripheral loss; however, these losses frequently go undetected because of the test characteristics of conventional perimetry.[20–23] We hypothesized that if sensitivity is already compromised to some degree due to loss of retinal ganglion cells, the increased visual task load could help uncover defects in the macular area and enable testing of visual function in a portable device, such as a computer tablet. In addition, by tapping into the low-level mechanisms of attention and visual perception, the test could predict performance on real-world tasks, which are often associated with low light and low contrast conditions[24,25], as well as high attentional demands caused by scene complexity.

The test has been incorporated into an iPad tablet (Apple computers, Inc., Cupertino, CA), which facilitates rapid and widespread acquisition, analysis, and dissemination of data. We present results of its evaluation for assessing functional deficits in glaucoma patients and for predicting risk of motor vehicle collisions and falls.

## Methods

Participants from this study were included in a prospective longitudinal study designed to evaluate functional impairment in glaucoma conducted at Visual Performance Laboratory of the Department of Ophthalmology at the University of California San Diego. The University of California San Diego Human Research Protection Program approved all the methods of this study. Written informed consent was obtained from all participants. All methods adhered to the tenets of the Declaration of Helsinki for research involving human subjects and the study was conducted in accordance with the regulations of the Health Insurance Portability and Accountability Act.

Subjects underwent a comprehensive ophthalmologic examination including review of medical history, visual acuity, contrast sensitivity assessment using the Pelli-Robson contrast sensitivity chart (Precision Vision, La Salle, IL), slit-lamp biomicroscopy, intraocular pressure (IOP) measurement, gonioscopy, dilated fundoscopic examination, stereoscopic optic disc photography, and SAP using the Swedish interactive threshold algorithm with 24–2 and macular 10–2 strategies (SITA Standard, Carl Zeiss Meditec, Inc., Dublin, CA, USA). Binocular visual acuity was measured using the Early Treatment Diabetic Retinopathy (ETDRS) chart and letter acuity was expressed as the logarithm of the minimum angle of resolution (logMAR). Only subjects with open angles on gonioscopy were included. Subjects were excluded if they presented with spherical refraction outside ±5.0 diopters or cylinder correction outside 3.0 diopters, or any other ocular or systemic disease that could affect the optic nerve or the visual field. All subjects also completed the Montreal Cognitive Assessment[26] to assess cognitive impairment. The Montreal Cognitive Assessment is a 30-point, ten-minute cognitive screening tool similar to the Mini-Mental State Examination, which involves assessment of short-term memory, visuospatial processing, executive function and higher level language ability.

### Participants

The study included 71 patients with glaucoma and 59 control subjects. Glaucoma was defined by the presence of repeatable abnormal SAP tests (pattern standard deviation with P <0.05 and/or a Glaucoma Hemifield Test outside normal limits) and corresponding optic nerve damage in at least one eye. Control subjects had no evidence of optic nerve damage and normal SAP visual field tests in both eyes. Only reliable visual field tests were included (less than 25% fixation losses and less than 15% false-positive errors). Optic nerve damage was assessed by masked grading of stereophotographs.

In order to evaluate binocular visual field loss, sensitivities of the monocular SAP threshold sensitivities of the right and left eyes were used to calculate an integrated binocular visual field, according to the binocular summation model described by Nelson-Quigg et al.[27] Mean sensitivities (MS) for the SAP 24–2 and SAP 10–2 exams were calculated as the average of all individual target threshold sensitivities of the binocular integrated field.

### The PERCEPT Visual Performance Test

The PERCEPT visual performance test was developed for the iPad with retina display (version 3) tablet in Objective-C using Apple iOS SDK 8.0 in the Xcode Integrated Development Environment (IDE). During initial development of the test, careful calibration was performed to ensure standardized conditions of testing, including fine control over the three main properties of the presented visual stimuli: size, timing, and contrast. In order to verify the stimulus duration timing, stimulus measurements were taken with a photo-resistor. A professional photometer (LiteMate PR–524 by Photo Research) mounted on a tripod was used to measure the light intensity of the presented stimuli, as well as of the background, and the different levels of contrast were rendered using alpha blending techniques. During calibration of the stimuli as well as during subsequent testing of patients, the auto-luminance option of the device was turned off and the luminance was set to maximum value. The tests were presented under standardized photopic ambient lighting conditions (85 candelas/m^2^). The viewing distance was 16 inches for all tests. In order to allow comparison of predictive ability for motor vehicle collisions and falls with UFOV results, the tests were performed binocularly, i.e., subjects with both eyes open.

**Fig 1:**
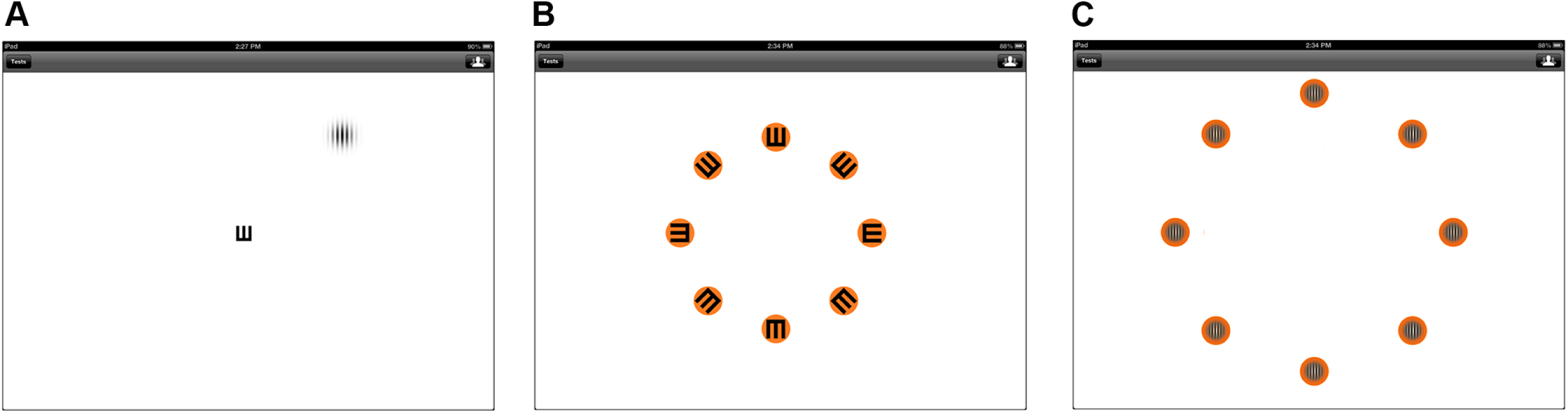
Screen shots taken during the PERformance CEntered Portable Test used for assessment of visual performance in glaucoma and control subjects. (A) Simultaneous presentation of the central and peripheral stimuli. (B) Response screen. The subject had to identify the orientation of the centrally presented “tumbling E”. (C) Response screen. The subject had to identify the location of the peripheral stimulus.

The test consisted of a dual task visual performance test. The central task consisted of recognizing the orientation of a “tumbling E” within a limited presentation time. The tumbling E was always presented at the center of the screen, with a size equivalent to 20/200 visual acuity, and it could assume 8 possible different orientations (Fig 1A). The peripheral target was a vertically oriented achromatic Gabor patch located at 7.7 degrees from central fixation in one of 8 different location meridians (Fig 1A). The Gabor patch itself subtended a visual angle of 2.71 degrees with a spatial frequency of 3.69 cycles per degree. The orientation of the Gabor patch was kept constant, however, the stimulus could assume one of 8 different positions. Patients were initially instructed to gaze at a small orange dot with a black rim in the center of the screen. This was then followed by presentation of the central tumbling E and the Gabor patch, which occurred simultaneously and for the same duration of time. The orientation of the tumbling E and the position of the Gabor patch were independent of each other. No further instructions were given as to whether they were allowed to make saccades while viewing the targets (tumbling E and Gabor patch).

Target presentation was then followed by a response screen with target orientation choices (Fig 1B). The subject had to identify the orientation of the central target (E) and the location of the peripheral target (Fig 1C) using the touchscreen. The tests progressed in a staircase fashion with decreasing presentation times. The initial presentation time of the tumbling E and the Gabor patch was 1000ms. As the patient successfully indicated the direction the tumbling E and the location of the Gabor patch, the duration of stimulus presentation decreased gradually from 1000ms to 16.67ms, which is the shortest possible duration on the iPad. Two successive correct answers were required for progression to the next level of difficulty. With three consecutive incorrect answers, the test was terminated and the shortest presentation time that the subject could correctly answer was recorded. The test was performed at 25% Weber contrast. The PERCEPT measured visual processing speed under a dual visual task at low contrast. In this study, we refer to its measurements simply as PERCEPT processing speed, measured in milliseconds (ms).

### Useful Field of View (UFOV)

The UFOV was also used to assess visual processing speed under divided attention (Subtest II, UFOV divided attention). The test has been described in detail elsewhere.[17,28] In brief, participants were asked to identify a cartoon representation of either a car or a truck (2 choices) that appeared in a box in the center of a 17-inch touchscreen monitor in addition to a concurrent peripheral localization task (an image of a car presented on one of eight radial spokes at a fixed eccentricity of approximately 11 degrees). The subject was then asked to identify the central target and to identify on which spoke the outside object was located. The test proceeded with decreasing presentation times, until the presentation time that would result in a 75% accurate response could be recorded. All stimulus presentations were at maximum contrast (100%).

### Driving records and history of falls

Driving records were obtained from all patients in the study from the California Department of Motor Vehicles (DMV). The history of involvement in motor vehicle collisions was gathered from the driving records and patients were subdivided into those with a positive history of motor vehicle collisions in the previous 3 years versus those without. Patients were also requested to answer a Driving Habits Questionnaire, contained selected items from the Manchester Driver Behavior Questionnaire.[29] The questionnaire contained an item requiring them to estimate the amount of miles driven throughout the week.

History of falls was also obtained using a standard questionnaire, the Falls Screening and Referral Algorithm.[30] The questionnaire asked about the number of falls the patient had over the past year, among other questions. These questionnaires have only been applied to the DIGS population since May 2014; therefore, only 97 of the 130 (75%) subjects had data on the number of falls. No statistically significant differences were seen in age, gender, PERCEPT processing speed and UFOV divided attention between subjects who completed the questionnaire versus those who did not (P>0.20 for all comparisons). All subjects had measurements of weight and height obtained at the time of testing. These were used to obtain the Body Mass Index (BMI) for each subject, as the quotient of mass (in kilograms) divided by the square of height (in meters). Subjects also completed a balance assessment on a force platform (ATMI, Danbury, CT). This was done to measure the somatosensory contribution to balance control, as this could be an important confounding factor in predicting risk of falls. Subjects were required to stand upright on the force platform for 120 seconds with ankles touching. Torque moments produced in the lateral and longitudinal direction around the center of foot pressure were measured. The test was done in a dark field, without any visual stimulation, by requiring the subjects to wear occluding goggles. Measurements obtained over the last 60 seconds were analyzed. The sum of the standard deviations (STD) of the two torque moments was calculated as a metric indicative of vision-independent postural stability (balance STD). Larger values of balance STD would be indicative of worse postural stability.

### Statistical Analyses

Normality assumption was assessed by inspection of histograms and using Shapiro-Wilk tests. Student *t*-tests were used for group comparison for normally distributed variables and Wilcoxon rank-sum test for continuous non-normal variables. Age-adjusted analyses were performed using analysis of covariance.

The receiver operating characteristic (ROC) curve was used to investigate the ability of the different tests in discriminating glaucomatous from control subjects. ROC curves were adjusted for age using an ROC regression model.[31] In addition, the ROC regression model was used to investigate the effect of disease severity on the performance of the tests in discriminating glaucoma from controls. This model has been described in detail previously by Medeiros et al.[31] Briefly, in the linear regression model

$$\mathrm{ROCx},x_{D}\;\left(q\right)=\Phi \;\left(\alpha_{1}+\alpha_{2}\Phi^{-1}\;\left(q\right)+\beta X+\beta_{D}X_{D}\right)$$

ROCx,x_D_ (q) is the sensitivity at the specificity of 1 –q, X represents the common covariates for healthy and glaucomatous eyes, such as age, and X_D_ represents disease-specific covariates such as disease severity. Φ is the normal cumulative distribution function. Coefficients α1 and α2 are the intercept and slope of the ROC curve. Once the ROC regression model is obtained, estimates of diagnostic accuracy can be obtained for specific values of disease severity, like in an ordinary regression approach. Confidence intervals for these estimates can be obtained using a bootstrap resampling procedure (*n* = 1000 resamples).

The ability to predict history of motor vehicle collisions was investigated using logistic regression models taking into account possible confounding variables such as age and average mileage driven per week.

The ability to predict history of falls was investigated with Poisson regression models, where the number of falls over the previous year was used as dependent variable and the PERCEPT and UFOV metrics were evaluated as independent variables. The models adjusted for age, gender, BMI, Montreal Cognitive Assessment score and average magnitude of displacement on the force platform. Vision may play a differential effect in maintaining balance in older compared to younger subjects, due to the changes in somatosensory function and patterns of activity with aging. Therefore, it is possible that a decline in visual function as measured by the tests in this study may have different predictive value for falls in older compared to younger adults. In order to evaluate this hypothesis, we included interaction terms in the regression model between age and the variables of interest (PERCEPT processing speed and UFOV divided attention), as well as between magnitude of postural stability and the variables of interest. Results of the Poisson models were given as the effect of the variables on the incident-rate ratio.

All statistical analyses were performed with commercially available software (Stata, version 13; StataCorp LP). The α level (type I error) was set at 0.05.

## Results

Table 1 shows demographic and clinical characteristics of the included patients. There were 71 patients in the glaucoma group and 59 in the control group with mean age of 70 ± 12 years and 61 ± 13 years, respectively (P<0.001). No significant differences were seen in race, gender or Montreal Cognitive Assessment score of cognitive impairment between the groups. Average worse-eye and better-eye SAP 24–2 mean deviation (MD) in glaucomatous eyes were -7.8 ± 7.4 dB and -4.0 ± 5.0 dB, respectively, with average binocular SAP 24–2 MS of 26.8 ± 3.4 dB. However, there was a very wide range of disease severity in the study, with SAP 24–2 MDs ranging from -28.9 dB to 1.6 dB in the glaucoma group.

**Table 1:** Demographic and clinical characteristics of the glaucomatous and control subjects who underwent visual performance testing with the Performance-Centered Portable Test and Useful Field of View.

	Glaucoma (n = 71)	Control (n = 59)	P-value
**Age (years)**	70 ± 12	61 ± 13	<0.001
**Gender, Female (%)**	32 (45%)	32 (54%)	0.298
**Race, Caucasian (%)**	50 (70%)	37 (63%)	0.122
**MOCA Score**	27.6 ± 2.7	27.8 ± 2.1	0.633
**Body-Mass Index (kg/m** ^**2**^ **)**	25.2 ± 4.2	26.5 ± 4.2	0.188
**Average magnitude of displacement on force platform (log mm)**	0.85 ± 0.16	0.88 ± 0.18	0.353
**Binocular visual acuity (logMAR)**	0.12 ± 0.14	0.08 ± 0.14	0.062
**Pelli-Robson Binocular contrast sensitivity (log)**	1.56 ± 0.19	1.69 ± 0.16	<0.001
**SAP 24–2 Binocular MS (dB)**	26.8 ± 3.4	30.3 ± 1.6	<0.001
**SAP 24–2 Worse-eye MD (dB)**	-7.8 ± 7.4	-0.7 ± 1.6	<0.001
**SAP 24–2 Better-eye MD (dB)**	-4.0 ± 5.0	0.2 ± 1.5	<0.001
**SAP 10–2 Binocular MS (dB)**	30.3 ± 5.0	33.8 ± 1.5	<0.001
**SAP 10–2 Worse-eye MD (dB)**	-7.3 ± 8.1	-0.6 ± 1.6	<0.001
**SAP 10–2 Better-eye MD (dB)**	-2.7 ± 4.7	0.1 ± 1.3	<0.001
**UFOV divided attention (ms)**	90 ± 100	51 ± 57	0.007
**PERCEPT processing speed (ms)**	493 ± 449	133 ± 198	<0.001

MOCA, Montreal Cognitive Assessment; SAP, standard automated perimetry; MS, mean sensitivity; MD, mean deviation; UFOV, Useful Field of View; PERCEPT, PERformance CEntered Portable Test.

**Fig 2:**
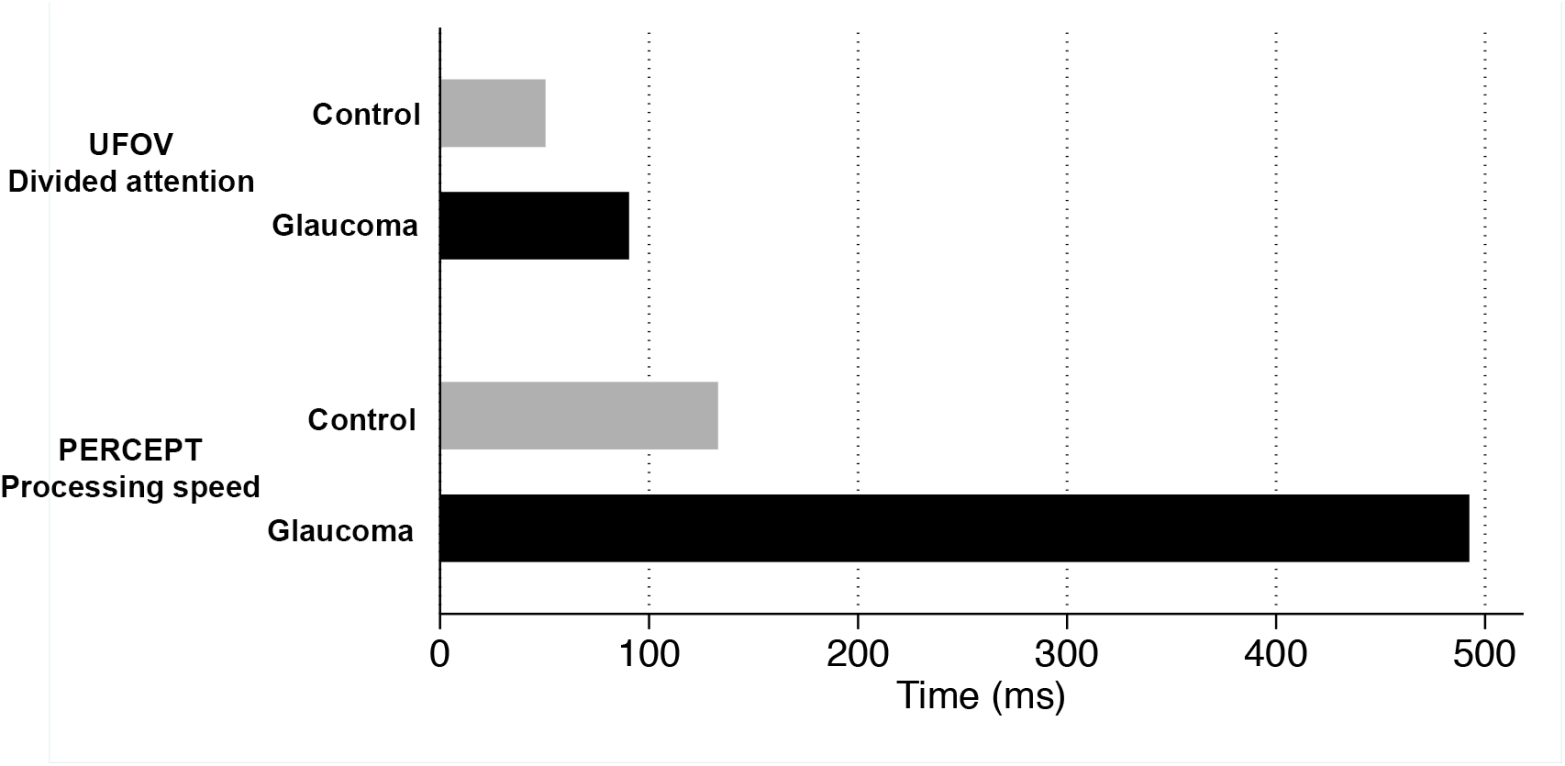
Bar graph illustrating the results of the PERformance CEntered Portable Test processing speed and Useful Field of View divided attention for assessment of visual performance glaucoma and control subjects.

Table 1 also summarizes the results of PERCEPT processing speed and UFOV divided attention. Patients with glaucoma had higher mean PERCEPT processing speed values than controls (493ms vs. 133ms, respectively; P<0.001), indicating a slower processing speed in the dual visual task at low contrast (Fig 2). Glaucoma patients also had significantly larger values of UFOV divided attention compared to controls (90ms vs. 51ms, respectively; P = 0.007). However, when adjusted for age, only the PERCEPT processing speed had significantly different results in glaucoma versus controls (mean age-adjusted difference: 243ms, P<0.001; analysis of covariance). UFOV divided attention was not significantly different between glaucoma and control subjects in age-adjusted analysis (mean age-adjusted difference: 17ms; P = 0.240; analysis of covariance).

**Fig 3:**
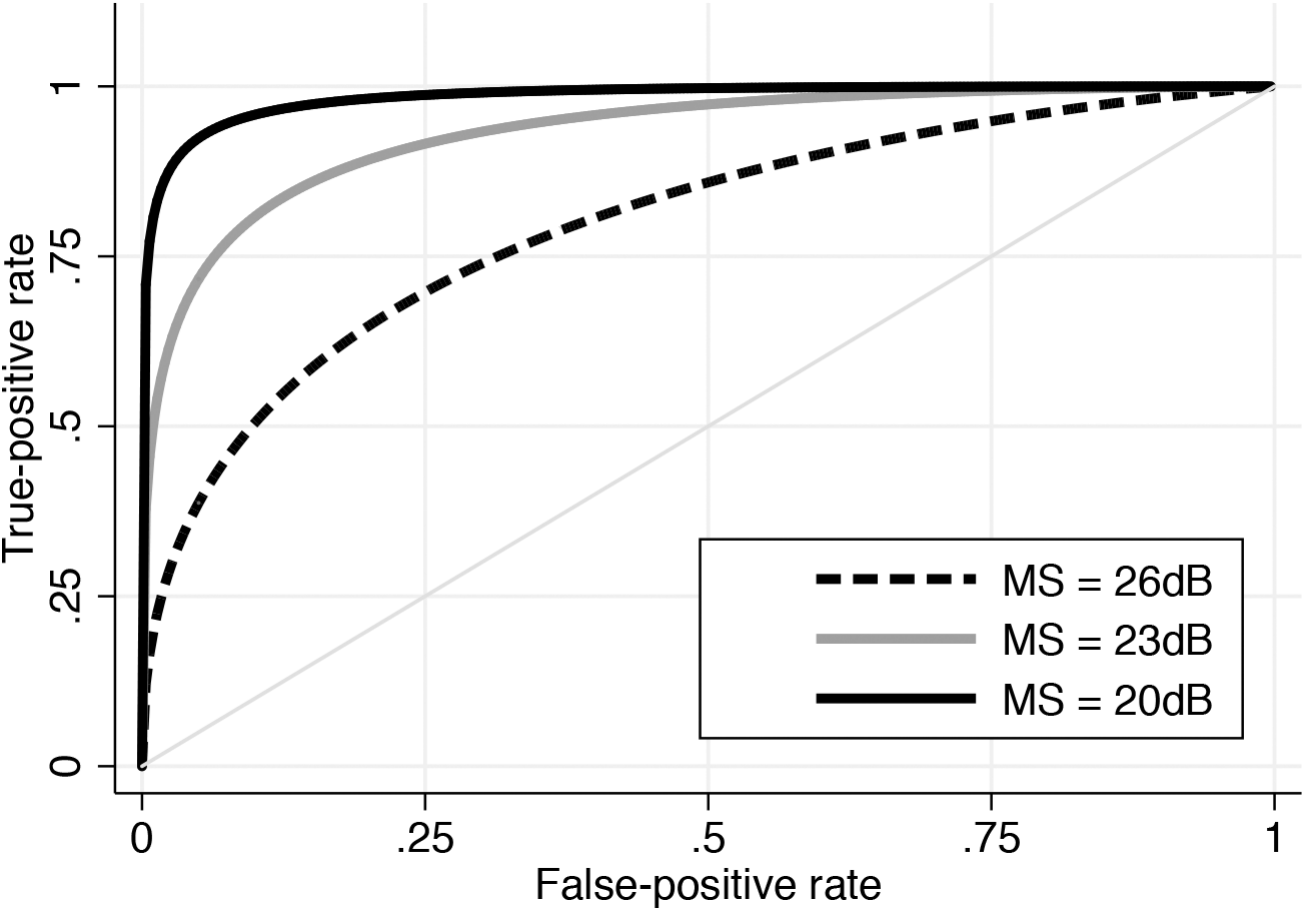
Receiver operating characteristic curves for the PERformance CEntered Portable Test processing speed parameter for discriminating glaucoma from control subjects, at arbitrary values of standard automated perimetry 24–2 binocular mean sensitivity.

Table 2 shows age-adjusted ROC curve areas for PERCEPT processing speed in discriminating glaucomatous from control subjects at different levels of disease severity, as determined by SAP 24–2 binocular MS. Sensitivities at specificities of 95% and 80% are also shown. As expected, substantial increases in discrimination were seen with increasing disease severity. For arbitrary values of SAP 24–2 binocular MS of 26dB, 23dB, and 20dB, ROC curve areas for the PERCEPT processing speed parameter were 0.80, 0.93, and 0.98, respectively (Fig 3). For specificity at 95%, the sensitivities of PERCEPT Performance-Centered portable test for detecting glaucomatous patients at these severities were 39%, 72% and 92%. For UFOV divided attention, ROC curve areas for corresponding disease severities were only 0.54, 0.63 and 0.72. At 95% specificity, sensitivities were 17%, 25% and 35%.

**Table 2:** Areas under the Receiver Operating Characteristic curves for the Performance-Centered Portable Test processing speed parameter for discriminating glaucomatous subjects from control individuals, at different levels of disease severity.

Disease severity (MS)	ROC curve area	Sensitivity at 80% specificity	Sensitivity at 95% specificity
**PERCEPT processing speed**			
**26 dB**	0.80 (0.67–0.90)	65% (49% - 83%)	39% (21% - 60%)
**23 dB**	0.92 (0.82–0.99)	87% (74% - 99%)	72% (43% - 94%)
**20 dB**	0.98 (0.91–1.00)	98% (87% - 100%)	92% (63% - 100%)
**UFOV divided attention**			
**26 dB**	0.54 (0.41–0.67)	33% (19% - 48%)	17% (6% - 32%)
**23 dB**	0.63 (0.45–0.78)	44% (24% - 64%)	25% (9% - 45%)
**20 dB**	0.72 (0.48–0.89)	55% (29% - 81%)	35% (12% - 65%)

ROC, receiver operating characteristic; MS, standard automated perimetry 24–2 mean sensitivity PERCEPT, PERformance CEntered Portable Test; UFOV, useful field of view; dB, decibels.

PERCEPT processing speed was significantly associated with SAP 24–2 binocular MS (R^2^ = 41%) and SAP 10–2 binocular MS (R^2^ = 30%). UFOV divided attention had weaker relationships compared to PERCEPT processing speed for both SAP 24–2 binocular MS (R^2^ = 19%; P <0.001 for comparison between tests) and SAP 10–2 binocular MS (R^2^ = 11%; P = 0.008 for comparison between tests).

**Fig 4:**
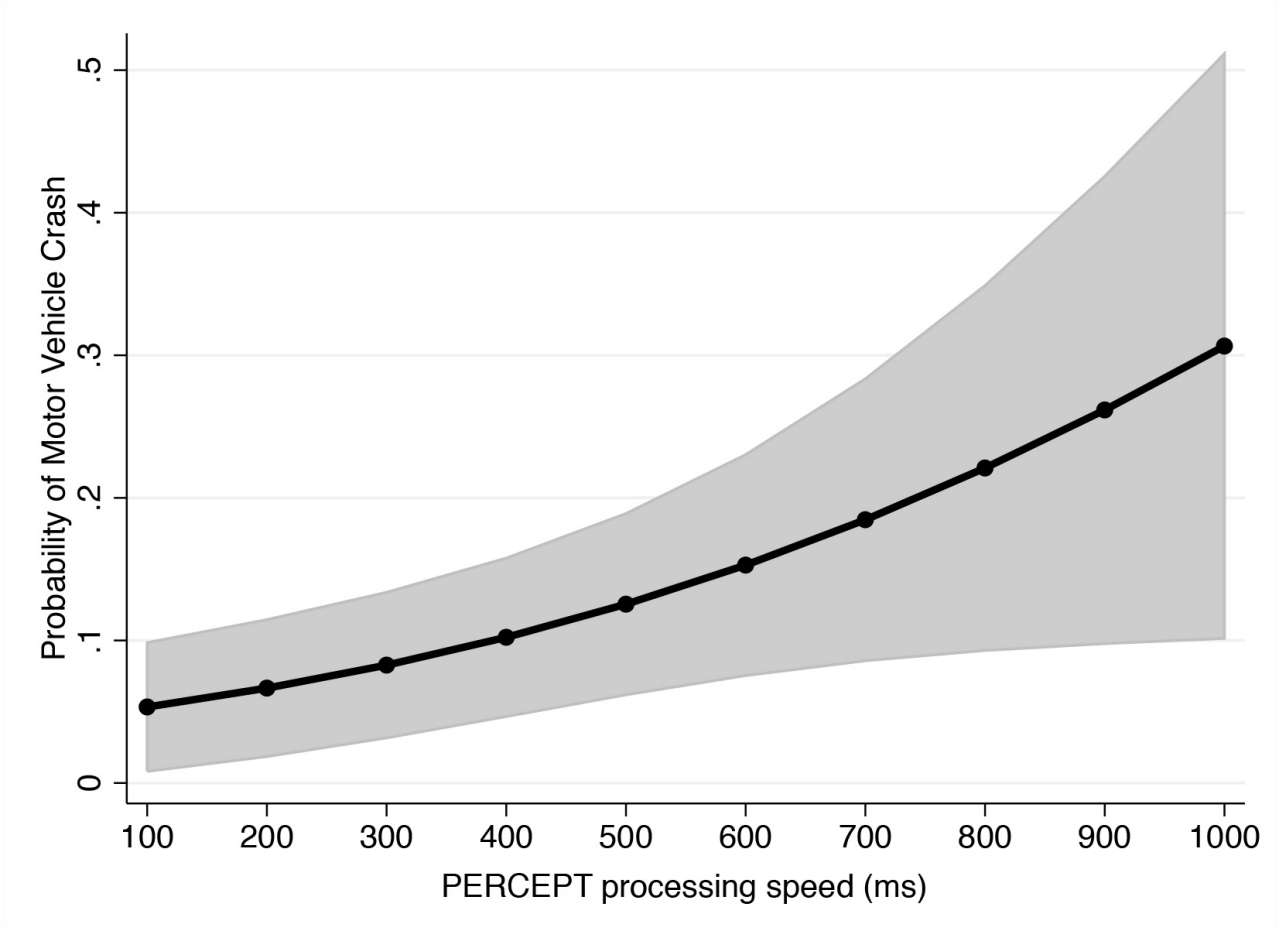
Relationship between probability of motor vehicle crash and results of the PERformance CEntered Portable Test processing speed parameter in the subjects who underwent visual performance assessment in the study. Lower processing speed values were associated with increasing probability of being involved in a motor vehicle crash.

From the 130 participants, 114 were active drivers (>10 miles/week). 10 of the 114 active drivers (9%) had history of motor vehicle collisions in the past 3 years obtained from the California DMV records. Patients with history of motor vehicle collisions had significantly higher average PERCEPT processing speed values compared to those without (664 ± 439 ms vs. 261 ± 360ms; P = 0.003). Average UFOV divided attention times were also higher in those with history of motor vehicle collisions versus those without, but the difference was not statistically significant (91 ± 101 ms vs. 65 ± 83 ms; P = 0.108). Table 3 shows the results of the multivariable model predicting motor vehicle collisions. After adjustment for age and average mileage driven per week, each 1 SD higher value of PERCEPT processing speed was associated with a 2.69 increase in the odds of motor vehicle collisions (odds ratio = 2.69; 95% CI: 1.40–5.15; P = 0.003). Fig 4 illustrates predicted risk of motor vehicle collisions for values of PERCEPT processing speed, adjusting for these confounding variables. After adjustment for the same confounding variables, UFOV divided attention was not significantly associated with risk of motor vehicle collisions (odds ratio = 1.24 per 1 SD higher; 95% CI: 0.72–2.13; P = 0.438). After adjustment for age and mileage driven per week, SAP 24–2 binocular mean sensitivity was significantly associated with history of motor vehicle collisions (odds ratio = 1.18 per 1dB lower, 95% CI: 1.04–1.33; P = 0.013).

**Table 3:** Results of the logistic multivariable model predicting history of motor vehicle crashes for subjects included in the study who underwent visual performance assessment with the Performance-Centered Portable Test processing speed parameter.

	Odds Ratio	95% CI	P-value
**PERCEPT PS, per 1 SD higher**	2.69	1.40–5.15	0.003
**Age, per decade older**	0.78	0.64–0.95	0.015
**Mileage per week, per 10 miles higher**	0.94	0.86–1.03	0.174

PERCEPT PS, PERformance CEntered Portable Test processing speed.

**Fig 5:**
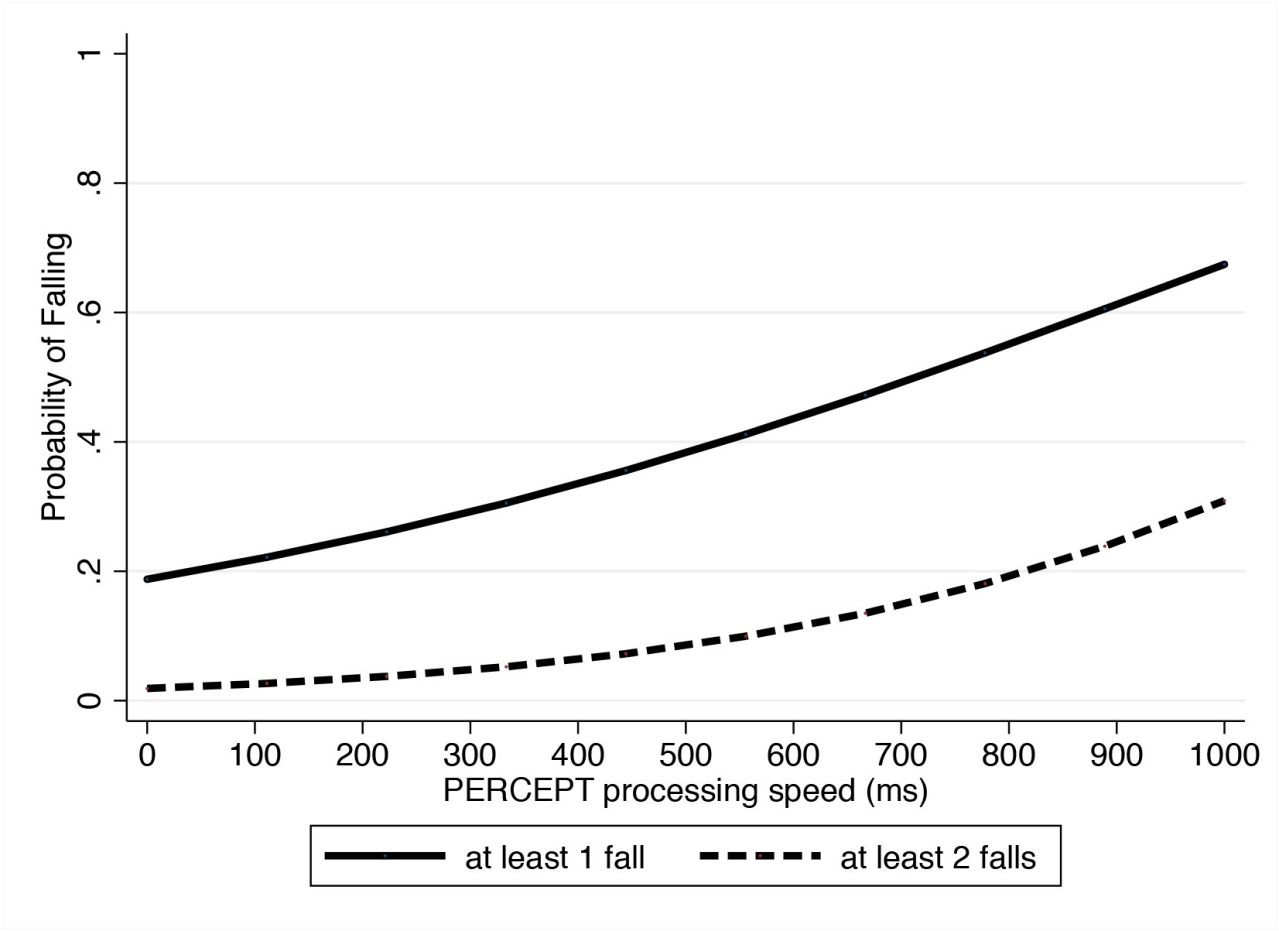
Relationship between probability of falling and results of the PERformance CEntered Portable Test processing speed parameter in the subjects who underwent visual performance assessment in the study. Lower processing speed values were associated with increased probability of falling.

Information about history of falls was available in 97 of the 130 participants. From the 97 subjects, 65 (67%) had no history of falls in the previous year, whereas 32 (33%) had at least 1 fall in the previous year. 13 subjects had 1 fall, 13 had 2 falls, 2 had 3 falls, 3 had 4 falls and 1 had 5 falls. Values of PERCEPT processing speed were predictive of history of falls in the multivariable Poisson regression model adjusting for confounding factors (Table 4). Each 1 SD increase in PERCEPT processing speed was associated with an incident-rate ratio of 1.95 (95% CI: 1.25–3.04; P = 0.003) (Table 3). However, there were significant interactions of PERCEPT processing speed with both age (P = 0.008) and balance STD on the force platform (P = 0.043). Fig 5 illustrates the relationship between the probability of falling and values of the PERCEPT processing speed parameter. UFOV divided attention was also predictive of falls. In the multivariable model, each 1 SD increase in UFOV divided attention had an incident-rate ratio of 1.68 (95% CI: 1.19–2.38; P = 0.003). For binocular SAP 24–2, each 1 dB lower mean sensitivity was associated with an incident-rate ratio of 1.15 (95% CI: 1.06–1.25; P = 0.001).

**Table 4:** Results of the Poisson regression model predicting history of falls for subjects included in the study who underwent visual performance assessment with the Performance-Centered Portable Test processing speed parameter.

	Incident-Rate Ratio (IRR)	95% CI	P-value
**PERCEPT processing speed, per 1 SD higher**	1.95	1.25–3.05	0.003
**Age, per decade older**	0.84	0.64–1.10	0.205
**PERCEPT processing speed x Age**	0.63	0.45–0.89	0.008
**Balance platform STD, per 1 log mm higher**	1.23	1.06–1.43	0.006
**PERCEPT processing speed x Balance platform STD**	0.84	0.71–0.99	0.043
**Gender, Female**	2.04	1.19–3.47	0.009
**Body Mass Index, per 1 kg/m** ^**2**^ **higher**	1.03	0.97–1.10	0.321

PERCEPT, PERformance CEntered Portable Test; STD, standard deviation of torque moments.

## Discussion

In the present study, we proposed and provided initial assessment of a test of visual performance in glaucoma that can be easily performed using a tablet computer. In this pilot assessment, PERCEPT processing speed was able to detect the presence of functional damage in a significant proportion of glaucoma patients and the results were significantly associated with history of falls and motor vehicle crashes in this population.

The ability of PERCEPT to identify functional loss in glaucoma seems to challenge the widely held view that the disease affects predominantly the peripheral visual field, as the iPad covered an area of less than 10 degrees from the fovea. However, glaucomatous damage to the macula has been demonstrated to be much more common than previously thought and it is possible that the characteristics of the PERCEPT test may facilitate detection of functional damage to this region. [20–23] The PERCEPT was developed in order to challenge the visual system by imposing a demanding central visual task (low contrast orientation task), while requiring simultaneous detection of a peripheral low contrast stimulus at increasingly shorter stimulus presentation times. In order to perform the central task correctly, individuals have to narrow their spotlight of attention. This contrasts with standard perimetry where no demanding central task is performed (neutral state of attention). Several studies have shown that once attention is focused in space, the unattended areas suffer a process of inhibition with decreased gain, which can make difficult the perception of visual stimuli outside the attended area.[32–34] Pestilli and Carrasco[32] have shown that sensitivity to contrast is substantially decreased in unattended areas, compared to a neutral condition. If detection of visual stimuli is already compromised to some degree due to loss of retinal ganglion cells in glaucoma, this challenging task could help uncover functional deficits.

An important finding of our study was that the PERCEPT metric was predictive of history of motor vehicle collisions, even after adjusting for confounding variables. Each 1SD higher value of this parameter (approximately 350ms) increased the odds of a motor vehicle collisions by almost 2.7 times. Although patients who had crashes also had longer times on the UFOV divided attention task, the UFOV was not predictive of history of motor vehicle collisions after adjusting for confounding factors. This result is in agreement with a recent population-based study by Friedman and colleagues[35] involving 2000 drivers. When adjustment was made for confounding variables, the relative motor vehicle collisions risk for subjects with UFOV results greater than 350ms compared to those with results less than 150ms was only 1.09 (95% CI: 0.73–1.61). As suggested by Friedman et al[35], differences compared to previous studies supporting the role of UFOV may be related to the lack of adjustment for important factors. The relatively small number of subjects with history of crashes in our study may also have precluded detection of significant effects with UFOV.

Both limited attentional resources as well as visual function deficits have been implicated in increasing risk of falls in elderly subjects.[36] PERCEPT processing speed and UFOV divided attention results were predictive of falls in our population, after adjustment for confounding factors. Importantly, as balance control depends on the complex relationship between sensory input from the visual, proprioceptive and vestibular systems, we obtained force platform data in a dark field in order to assess and adjust our results for non-visual components contributing to balance. A significant interaction was seen between force platform results and PERCEPT processing speed, suggesting that vision may play a different role in predicting falls depending on the strength of the somatosensory contribution to balance.

The superior performance of the PERCEPT compared to UFOV in detecting functional loss in glaucoma may also be attributed to the different characteristics of the tests. The central task in the PERCEPT is considerably more demanding than that of UFOV. While the central task in UFOV requires patients to simply distinguish between a representation of a car or truck in a 2-alternative forced choice test, the PERCEPT central task consisted of an orientation task in an 8- alternative forced choice test test. As another difference, UFOV testing is performed at maximum contrast, while the PERCEPT testing uses low contrast. It should be noted, however, that both UFOV and PERCEPT results might also be affected by conditions other than glaucoma that could be associated with slow visual processing speed and this needs to be considered when interpreting their results.

Our study has limitations. PERCEPT testing was performed under controlled situations, such as room illumination and at fixed distance of the subject from the screen. Although such testing conditions can be easily replicated in other environments, it will be important to assess the effect of these testing conditions in other scenarios and evaluate the predictive value and reproducibility in other populations. Also, it is possible that for some longer presentations, saccadic eye movements may have occurred, which could have underestimated the degree of impairment for some subjects. However, this would likely lead to a decrease in the differences found between glaucomatous and healthy subjects. Our analyses did not include evaluation for coexisting morbidities such as depression, for example, which could potentially affect test results and the outcome variables. In addition, patients with glaucoma may exhibit deficits in eye-hand coordination,[37] which could perhaps affect the results of the test. Longitudinal prospective studies should be conducted to evaluate these issues. As another limitation, driving records from the California DMV did not contain information of whether subjects were at-fault in the motor vehicle collisions. However, one would expect that including subjects who were not at-fault would actually tend to decrease the chance of finding a significant result. Although we adjusted our analyses for mileage driven per week, we were not able to obtain detailed history of driving exposure and this should also be subject of future studies with larger samples.

It could also be argued that some of the subjects would have abnormal results on the PERCEPT testing from loss of contrast sensitivity due to coexisting media opacities, such as cataract. We conducted a subgroup analysis including only subjects who were pseudophakic in both eyes (20 glaucomatous and 14 controls). Despite the relatively small sample available for this analysis, the PERCEPT processing speed was still significantly higher in glaucomatous compared to controls (828 ± 319ms vs. 256 ± 269; P<0.001). It should also be noted that even though cataract or other media opacities could influence results for both PERCEPT and UFOV, this would not preclude their use for assessment of visual impairment.

In conclusion, a portable platform for testing visual function was able to detect functional deficits in glaucoma and its results were associated with history of falls and motor vehicle collisions in this population. Our results suggest that strategies for evaluation of functional impairment in glaucoma using portable platforms may be feasible. However, further studies are necessary to assess its validity in other populations.

## Supporting Information

S1 Datafile Minimal dataset used in the study. Dataset including PERCEPT and UFOV metrics.
(XLS)
